# In-Depth Characterization and Functional Analysis of Clonal Variants in a *Mycobacterium tuberculosis* Strain Prone to Microevolution

**DOI:** 10.3389/fmicb.2017.00694

**Published:** 2017-04-24

**Authors:** Yurena Navarro, Laura Pérez-Lago, Marta Herranz, Olalla Sierra, Iñaki Comas, Javier Sicilia, Emilio Bouza, Darío García de Viedma

**Affiliations:** ^1^Servicio Microbiología Clínica y Enfermedades Infecciosas, Hospital General Universitario Gregorio MarañónMadrid, Spain; ^2^Instituto de Investigación Sanitaria Gregorio MarañónMadrid, Spain; ^3^CIBER Enfermedades Respiratorias, CIBERESMadrid, Spain; ^4^CEI Campus Moncloa, UCM-UPMMadrid, Spain; ^5^Centro de Vigilancia Sanitaria Veterinaria, Universidad Complutense MadridMadrid, Spain; ^6^Unidad Mixta Genómica y Salud, Centro Superior de Investigación en Salud Pública (FISABIO)-Universitat de ValènciaValencia, Spain; ^7^CIBER en Epidemiología y Salud PúblicaMadrid, Spain; ^8^Unidad de Medicina y Cirugía Experimental, Hospital General Universitario Gregorio MarañónMadrid, Spain; ^9^Departamento de Medicina, Facultad de Medicina, Universidad Complutense de MadridMadrid, Spain

**Keywords:** microevolution, *Mycobacterium tuberculosis*, functional analysis, *in vitro* infections, *in vivo* infections, whole genome sequencing

## Abstract

The role of clonal complexity has gradually been accepted in infection by *Mycobacterium tuberculosis* (MTB), although analyses of this issue are limited. We performed an in-depth study of a case of recurrent MTB infection by integrating genotyping, whole genome sequencing, analysis of gene expression and infectivity in *in vitro* and *in vivo* models. Four different clonal variants were identified from independent intrapatient evolutionary branches. One of the single-nucleotide polymorphisms in the variants mapped in *mce*3R, which encodes a repressor of an operon involved in virulence, and affected expression of the operon. Competitive *in vivo* and *in vitro* co-infection assays revealed higher infective efficiency for one of the clonal variants. A new clonal variant, which had not been observed in the clinical isolates, emerged in the infection assays and showed higher fitness than its parental strain. The analysis of other patients involved in the same transmission cluster revealed new clonal variants acquired through novel evolutionary routes, indicating a high tendency toward microevolution in some strains that is not host-dependent. Our study highlights the need for integration of various approaches to advance our knowledge of the role and significance of microevolution in tuberculosis.

## Introduction

The idea that *Mycobacterium tuberculosis* (MTB) infection of a single case is caused by a single strain is increasingly debated. Genotyping has enabled us to describe cases of co-infection by ≥2 different strains (mixed infection) and coexistence of clonal variants of the same strain (polyclonal infection) (García de Viedma et al., 2005; Shamputa et al., 2006; Al-Hajoj et al., 2010; Navarro et al., 2011; Huyen et al., 2012; Muwonge et al., 2013). In the latter, clonal variants emerge through microevolution phenomena, which make it possible to detect subtle changes when applying standard fingerprinting strategies (IS*6110*-RFLP or mycobacterial interspersed repetitive units–variable number of tandem repeats [MIRU-VNTR]). These changes can have a functional impact on the expression of neighboring genes and can increase the variability of antigenic proteins (Akhtar et al., 2009; Olsen et al., 2009; Yindeeyoungyeon et al., 2009; Tantivitayakul et al., 2010; Perez-Lago et al., 2011, 2013).

Most studies on microevolution in infection by MTB or on the functional characterization of MTB clonal variants merely describe the genotypic changes involved. If they include a functional analysis, then this is only partially based on gene expression assays or standard infection models. In our study, we attempted to overcome this fragmented view of clonal complexity of MTB infection by performing an in-depth analysis of a representative case of clonally complex MTB infection by integrating the following approaches: (i) complete characterization of the variants by both standard genotyping and whole genome sequencing (WGS); (ii) gene expression analysis; and (iii) evaluation of infectivity by analyzing the behavior of clonal variants not only in standard infections, but also in co-infections in broader experimental conditions than usual, i.e., applying both cellular and animal models. We show how integrating various approaches can advance our knowledge of the role and significance of microevolution in MTB infection.

## Materials and methods

### *Mycobacterium tuberculosis* clonal variants

The clonal variants (A and B) were isolated from a patient with recurrent tuberculosis and identified using 15-locus MIRU-VNTR, as described in Martín et al. (2011).

### Expanded characterization of the clonal variants

#### 24-locus MIRU-VNTR

Clonal variants were further characterized using 24-locus MIRU-VNTR (Oelemann et al., 2007), as described in Navarro et al. (2011), and IS*6110*-restriction fragment length polymorphism (RFLP), following international standardization guidelines (van Embden et al., 1993).

#### Ligation-mediated PCR (LM-PCR)

IS*6110* sequences were mapped using LM-PCR, as described in Perez-Lago et al. (2011).

#### Alelle-specific-PCR (ASO-PCR)

Alelle-specific PCRs were designed to analyze single colonies to assign the alelles present for several SNPs identified in the following Rvs: Rv1201, 1527, 1553, 1963, 2209, 2579.

The reactions were carried out in 50 μl including MgCl_2_ (1.2, 0.9, 1.5, 1.3, 1.3, and 1.3 mM; for the different SNPs in the order indicated above), 0.4 μl dNTPs (10 μM); 0.5 μl DMSO, 1.5 μl s of each primer (Supplementary Table; 10 μM), 0.4 μl AmpliTaq Gold enzyme. The PCR run was constituted by 30 rounds of the cycle: 10′ 95°C, 1′ 95°C, 1′Tm (64, 60, 63, 64, 64, 64°C, for the different SNPs in the order indicated above), 10′ 72°C and a final tail of 4°C.

For each SNP, two independent PCRs were run. The first one using the selective primer complementary for the allele in the SNP and, as a control, a second PCR targeting the complementary allele. The expected sizes for the products were 239, 402, 239, 207, 234, and 147 bp (for the different SNPs in the order indicated above).

### Whole genome sequencing

WGS was performed as detailed elsewhere (Perez-Lago et al., 2014) using a HiSeq 2000 device and a Miseq device (Illumina), which generated 101-51–bp paired-end reads. We mapped the reads for each strain using the Burrows-Wheeler Aligner and the ancestral MTB genome as detailed elsewhere (Comas et al., 2013). SNP calls were made with SAMtools and VarScan (coverage of at least 10×, mean SNP mapping quality of 20). The genome was compared between strains using an in-house script written in R.

### Gene expression assay

Relative quantification assays based on reverse transcriptase polymerase chain reaction (RT-PCR) were performed to examine expression of the gene involved in the microevolution events. The clonal variants were grown in 7H9 liquid medium (Difco) supplemented with 10% ADC (Becton Dickinson) and 1% Tween 80 (Merck) for 3 weeks. Cell lysis and RNA extraction were performed as previously described (Perez-Lago et al., 2013). RNA was reversed-transcribed using the High Capacity RNA-to-cDNA Kit (Invitrogen, Life Technologies, CA, USA). This step was followed by qRT-PCR amplification (preincubation at 95°C for 10 min and 45 cycles of 95°C for 10 s, 60°C for 10 s, and 72°C for 20 s) using the LightCycler® FastStart DNA Master SYBR Green I kit (Roche) and the primers FyrbE3A (5′-GGTGTTTCTCATGCACGTCT-3′) and RyrbE3A (5′-CCGACCGACATGCCCTTATA-3′). The results of the assay were expressed as the ratio of the values obtained for the variant harboring the SNP to the values from the wild-type variant. Three independent quantitative RT-PCR assays were performed using 2 independent RNA extractions. A 1-sample *t*-test was used to determine whether the average expression ratio was statistically different from 1 (*p* < 0.05).

### Infection assays

#### In vivo infection model

Specific pathogen–free 8-week-old female Balb/c mice were obtained from Charles River Laboratories (L'Arbresle, France) and from our animal experimental laboratory (Instituto de Investigación Sanitaria Gregorio Marañón, Madrid, Spain). The mice were shipped under appropriate conditions, with the corresponding certificate of health and origin. All the animals were kept under controlled conditions in a biosafety level 3 facility with food and water *ad libitum*.

Mice were anesthetized by intraperitoneal injection with xylazine (0.75 mg/g) and ketamine (0.1 mg/g) and subsequently infected with 200 μl of inoculum (1–5 × 10^5^ bacilli) by intravenous inoculation in the lateral tail vein.

Monitoring of infections by each clonal variant was based on colony forming unit (CFU) counts. The lungs and spleen of the infected mice were individually homogenized in PBS supplemented with 0.05% Tween 80. Serial 10-fold dilutions of the homogenate were plated on Middlebrook 7H11 agar (Difco). The growth rates for each clonal variant were calculated by linear regression of the CFU logarithm. Eighteen mice were infected, and 3 mice were analyzed at each point for each strain (day 1, week 1, and week 3).

The results of the assays in which the mice were simultaneously co-infected with the 2 clonal variants were analyzed by comparing the proportion of co-infecting strains before infection (adjusting to a 1:1 proportion) and after infection. The lungs and spleen of the infected mice were individually homogenized and plated at each time point (day 1 and week 5) to analyze the allelic value of locus MIRU 42 in 40 single colonies. Ten mice were co-infected, and standard deviations were calculated on the basis of the results obtained from 5 mice at each time point.

#### *In vitro* infection model

THP-1 cells were differentiated to macrophages and simultaneously co-infected with both clonal variants following the protocol described by Alonso et al. (2010) at a multiplicity of infection of 3 bacteria per cell. The proportion of co-infecting variants before and after infection was calculated at 3 h and day 7 by plating 10-fold serial dilutions of lysates on Middlebrook 7H11 agar (Difco). Seventy colonies were analyzed at each time point using simplex PCR of locus MIRU 42.

### Fitness assay

Clonal variants were subcultured on Mycobacteria Growth Indicator Tubes (BACTEC MGIT 960 System; Becton Dickinson) supplemented with BBL™ PANTA™ and BACTEC MGIT 960 Growth Supplement, as indicated by the manufacturer. Inocula were obtained from positive tubes (3 days) following the protocol used for antimicrobial susceptibility testing. Three MGITs were inoculated after ensuring equivalent bacterial concentrations in the 3 aliquots of inoculum used by plating them on Middlebrook 7H11 agar (Difco). Growth curves were obtained by monitoring the growth units (GU) every hour using BD EpiCenter™. The fitness of the clonal variants was compared based on 2 parameters taken from the growth curves: (i) lag phase (time to a positive threshold [75 GU]) and (ii) rate growth (time required for the 4,000–6,000–GU increase).

The means and standard deviations were determined, and one-way analysis of variance with repeated measures was used to determine *P*-values, which were adjusted using a Bonferroni correction.

## Results

### Complete characterization of clonal variants

**Figure 1:**
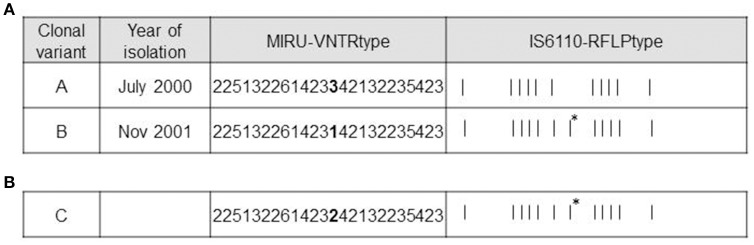
**Genotypic features of clonal variants A, B, and C. (A)** Genotypes of variants A and B. MIRU-VNTR loci differing between the variants are highlighted in bold. The asterisks indicate the additional IS*6110* band in variant B. **(B)** Genotypes of variant C. MIRU-VNTR loci differing between variants A and B are highlighted in bold. The asterisk indicates the same additional IS*6110* band obtained from variant B.

Four isolates from the patient with recurrent infection were available for analysis, 1 for the first episode (July 2000) and 3 for the second one (August-November 2001). Available genotyping data for the clonal variants using 15-locus MIRU-VNTR were further analyzed using 24-locus MIRU-VNTR. Differences in the VNTR analysis were restricted to a single-locus variant in locus MIRU 42 (424/Mtub04) (3 repetitions in the first isolate [VNTR variant A] and 1 repetition in the last isolate [VNTR variant B]) (Figure 1A). A mixture of VNTR variants A and B (detected by the observations of the corresponding double alleles in MIRU 42) was detected in the 2 intermediate specimens (August), thus leading us to select the first and last isolates for further genotyping.

According to RFLP, the differential IS*6110* band detected in one of the 2 variants (Figure 1A) was localized using LM-PCR and mapped at the location described for one of the 16 insertions of H37Rv (3551227-3552587).

WGS revealed 11 different SNPs between the 2 variants. After comparing the WGS data with the most recent common ancestor of MTB as a reference, we obtained 6 specific SNPs from variant A and 5 from variant B (Table 1). Of the 6 specific SNPs from variant A, 3 were non-synonymous and 3 were synonymous. Of the 5 SNPs from variant B, 4 were non-synonymous, and the remaining one was intergenic.

**Table 1:** **Specific SNPs from variants obtained by WGS**.

**Variant A**	**Ancestor**	**S/NS/1**	**Gene**
T	C	NS	Rv1963c (*mce3R*)
C	A	NS	Rv2579 (*dhaA*)
T	C	NS	Rv2921c (*ftsY*)
T	C	s	Rv0836c
G	T	s	Rv1487
G	C	s	Rv1497 (*lipL*)
**Variant B**	**Ancestor**	**S/NS/1**	**Gene**
T	C	NS	Rv1201c
T	G	NS	Rv1527c (*pks5*)
A	G	NS	Rv1553 (*frdB*)
G	C	NS	Rv2209
C	G	I	Rv2644c–Rv2645

*S, synonymous SNP; NS, Non-synonymous SNP; l, lntergenic SNP*.

### Analysis of functional relevance

The next step in the characterization of these clonal variants was to determine the functional relevance of the subtle modifications identified. The IS*6110* variation mapped in a hotspot for the IS*6110* insertion sequence, thus minimizing the potential functional significance of this variation. The MIRU-VNTR modifications mapped in a region between 2 stop codons for 2 adjacent genes (Rv0353 and Rv0354), again reducing the likelihood of a functional impact for these changes. In contrast, the fact that some of the SNP-based variability revealed by WGS corresponded to non-synonymous changes in relevant genes made it worthwhile to evaluate their functional impact.

**Figure 2:**
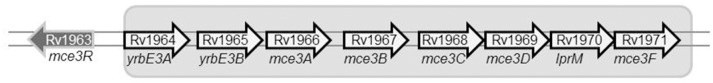
**Diagram showing the location of the gene *mce*3R within the *mce*3R operon**.

We evaluated the potential impact of SNP-based variability by focusing on the SNP in Rv1963c (*mce3R*), because the SNP maps in a repressor of several genes included in the yrbE3A-Rv1971 operon (Santangelo et al., 2008; Figure 2). We compared the expression of the first gene in the operon, *yrbE3A*, in the 2 variants. A 0.7910-fold ratio (0.6936–0.8884; *p* < 0.05) was observed in the expression of *yrbE3A* in variant A compared with variant B, indicating that the SNP detected in variant A increased the efficiency of the repressor Mce3R.

### Infectivity of clonal variants

The infectivity of the clonal variants was measured in both murine and cellular models. In the murine model, no significant differences were found in the growth rates of each variant in the lung (0.8039 ± 0.1707 and 0.8658 ± 0.1683 for variants A and B) or in the spleen (0.2328 ± 0.2996 and 0.5926 ± 0.1911 for variants A and B).

**Figure 3:**
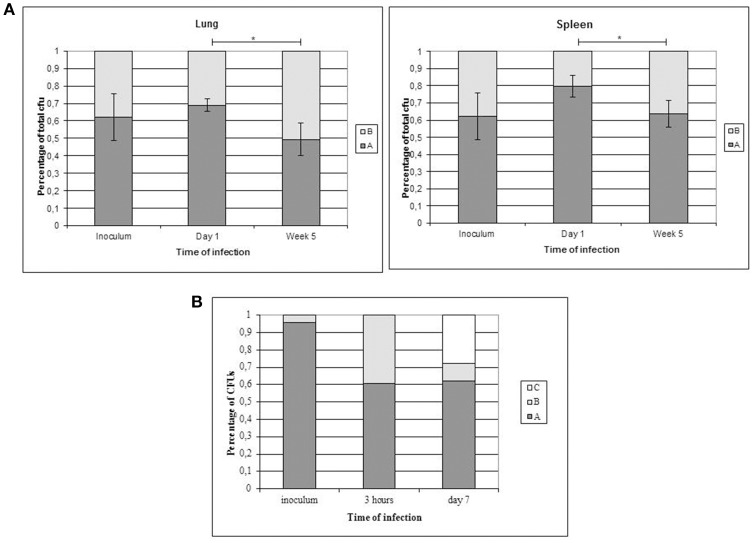
**Co-infection assays with both clonal variants**. Asterisks indicate statistical significance (*p* < 0.05). **(A)** Proportion of co-infecting variants obtained in the lung (left panel) and spleen (right panel) from 5 mice at each time point. **(B)** Co-infection of THP-1 cells with the clonal variants.

To evaluate the presence of subtle differences between the infectivity of the variants that were not revealed in standard infection assays, we performed competitive assays by simultaneously infecting mice with the 2 clonal variants. We did not detect differences in the proportion of the variants at day 1 compared with the proportion in the inoculum (both in lung and in spleen Figure 3A). However, the representativeness of variant B between day 1 and week 5 increased both in the lung (from 0.3095 ± 0.0336 at day 1 to 0.5058 ± 0.0950 at week 5) and in the spleen (from 0.2019 ± 0.0624 at day 1 to 0.3638 ± 0.0779 at week 5) (*p* < 0.05) (Figure 3A).

In order to obtain new evidence of the likely higher infectivity of clonal variant B, we performed a new co-infection experiment. On this occasion, we used macrophages and modified the inoculum to force underrepresentation of clonal variant B (96:4). Even at this unbalanced proportion, more efficient uptake was observed for variant B than for variant A, meaning that the 96:4 proportion in the inoculum became 61:39 at 3 h (Figure 3B). Measurements could not be taken appropriately at day 7 owing to an unexpected finding, namely, the emergence of a previously undetected clonal variant (variant C: 2 repetitions at locus MIRU 42, Figure 1B). The 3 variants were now found at a proportion 62:10:28 (A:B:C). Consequently, the representativeness of variant A remained constant compared with that observed at 3 h after infection, whereas the presence of variant B decreased.

### Characterization of the new clonal variant

MIRU-VNTR analysis indicated that variant C only harbored a difference in locus MIRU 42 (allelic value 2). The IS*6110*-RFLP type was identical to that of clonal variant B, suggesting that variant B was its parental strain (Figure 1B). WGS of the new variant revealed no SNPs with respect to variant B, confirming that variant C derived from variant B.

**Figure 4:**
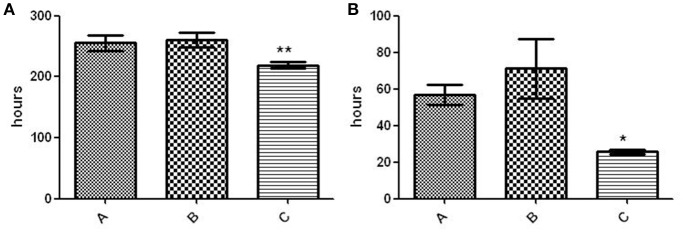
**Fitness assay of the 3 variants**. **(A)** Lag phase (time to positive threshold [75 GU]). The asterisks indicate statistical significance (*p* < 0.01). **(B)** Growth rate (time required for the 4,000–6,000–GU increase). The asterisk indicates statistical significance (*p* < 0.05).

The fitness of variant C was compared with that of the other 2 variants and proved to be higher, as indicated by its shorter lag phase (219 ± 5.292 h vs. 260 ± 12.12 h [variant B] and 255.3 ± 12.5 [variant A]; *p* < 0.01) (Figure 4A) and rate of growth (25.62 ± 1.22 vs. 71.23 ± 16.32 h [variant B] and 56.84 ± 5.569 h [variant A]; *p* < 0.05) (Figure 4B).

### Analysis of intrapatient microevolution dynamics

In order to determine the dynamics by which variant A (July 2000) was replaced by variant B (November 2001), we recovered the 2 intermediate specimens (August 2001, specimens 1 and 2) in which the 2 variants coexisted to evaluate whether it was possible to track the progressive elimination of variant A. Taking advantage of the differential SNPs found between the 2 variants, we selected the seven non-synonymous SNPs to assess the representativeness of each variant by analyzing multiple single colonies. For this aim we tailored allele specific PCRs to directly track the two alternative alleles in the seven SNPs. The ASO-PCR targeted to the analysis of the SNP in Rv2921 could not be optimized. The analysis of the remaining six SNPs on 30 colonies (12 and 18 from specimens 1 and 2, respectively) revealed that in the specimen 1 coexisted the variant A with two novel variants (new variant 1 and 2), sharing 4 and five alleles out of six, respectively, with variant B (Table 2). In the specimen 2 we observed the coexistence of variant A with the new variant 2.

**Table 2:** **Distribution of SNPs (in bold, after comparing to the ancestor reference) identified in the clonal variants by analyzing single colonies**.

		**Episode 1**	**Episode 2**					
		**July 2000**	**August 2001:Specimen 1 (12 colonies)**			**August 2001:Specimen 2 (20 colonies)**		**November 2001**
**SNP:Gene**	**Ancestor**	**Variant A**	**Variant A (3)**	**New variant 1 (2)**	**New variant 2 (7)**	**Variant A (6)**	**New variant 2 (14)**	**Variant B**
SNP1:Rv1201	G	G	G	**T**	**T**	G	**T**	**T**
SNP2:Rv1527	G	G	G	**A**	**A**	G	**A**	**A**
SNP3:Rv1553	C	C	C	C	**G**	C	**G**	**G**
SNP4:Rv1963	C	**T**	**T**	C	C	**T**	C	C
SNPS:Rv2209	G	G	G	G	G	G	G	**C**
SNP6:Rv2579	A	**C**	**C**	A	A	**C**	A	A
SNP7:Rv2921	C	**T**						C

*The number between brackets indicates the number of colonies in which that composition of SNPs was identified*.

**Figure 5:**
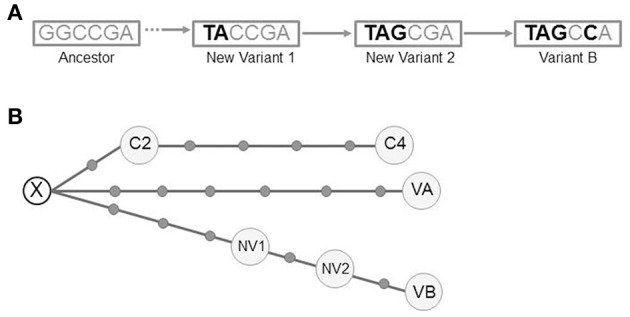
**(A)** Schematic representation of the pathway of SNP acquisition for variant B. The dashed arrow indicates the lack of information to determine the potential intermediates between the ancestor and the new variant 1. **(B)** Median-joining network for all the variants from the 3 clustered cases (NETWORK 5.0). The circles correspond to the different variants (VA, VB, NV1, NV2, C2, and C4). Each dot along the lines linking the circles corresponds to a single-nucleotide polymorphism detected between the linked variants. A hypothetical unsampled variant (X) is postulated by the algorithm. VA (variant A), VB (variant B), NV1 (New variant 1), NV2 (New variant 2), C2 (clustered case 2), C4 (clustered case 4).

The detailed analysis of the intermediate isolates allowed us to rule out our firstly assumed hypothesis of a chronological substitution of variant A by variant B. Instead, there was a coexistence of new evolutionary intermediates acquiring sequentially the variant B alelles (Figure 5A). In summary we could identify 4 independent clonal variants involved at different stages of the microevolutionary process along the infection.

### Analysis of interpatient microevolution dynamics in clustered patients

Our final step was to search for other cases in the population involved in a transmission cluster with the case we report. We searched for cases infected by each of the initial and final variants A and B. For variant A, we identified 2 cases (C2 and C4, years 2003 and 2004 respectively) and for variant B none.

We performed WGS analysis with these 2 new isolates. Neither shared the alleles found in the SNPs of variants A and B with respect to the ancestor, indicating that they had followed an independent microevolutionary branch (Figure 5B) from a common unsampled node (X). C2 and C4 were sequential steps of that branch. The SNP acquired in C2 was synonymous and mapped in Rv0037 (conserved membrane protein). Three of the 4 SNPs from C4 mapped in Rv0015 (non-synonymous, transmembrane serine/threonine-protein kinase A), Rv1821 (synonymous, preprotein translocase ATPase secA2), and Rv2934 (non-synonymous, phenolpthiocerol synthesis type-I polyketide synthase). The fourth SNP was intergenic.

## Discussion

We performed an in-depth study of the clonal variants that emerged in a patient with recurrent tuberculosis. Initial genotypic characterization confirmed that the strains isolated from each episode were clonal variants because they only differed in a single-locus variant in 24 MIRU loci and in a single IS*6110* copy. Similarly subtle differences have been reported for clonal variants in various studies (Shamputa et al., 2006; Al-Hajoj et al., 2010; Navarro et al., 2011).

WGS was applied to obtain a more exhaustive description of the degree of variability between the clonal variants, and 11 SNPs were identified. These figures are higher than expected according to the variability observed in transmission chains (similarity threshold of 12 SNPs before isolates can be considered clustered; Walker et al., 2013). Our findings indicate that intrapatient variability itself could be close to this threshold. Also, given previously reported mutation rates in MTB, usually within the range of 0.25–0.5 SNPs per genome per year, the accumulation of SNPs in the intrapatient variants is much higher than expected. It suggests a higher tendency to acquire variability by this strain, but we must also leave open the possibility that part of that variability could have been acquired during the latency period, as it has been suggested elsewhere (Lillebaek et al., 2016).

We compared the alleles found in the 11 SNPs with those found in the most recent common ancestor of MTB as a reference (Comas et al., 2010, 2013) and found that variant B did not evolve directly from variant A, because 6 and 5 alleles found in the 11 SNPs were specific to variants A and B, respectively. Therefore, each variant represented an independent evolutionary path from a common parental strain that had not been sampled in the specimens analyzed. The results obtained from this new analysis fitted better with the consensus variability thresholds (Walker et al., 2013), thus illustrating that it is essential to integrate allelic data from the ancestor in order to interpret SNP data appropriately and that this step is needed to establish the true phylogenetic relationships between clonal variants.

Our naïve initial interpretation of 2 intrapatient variants emerging one from the other became a more complex picture of 2 independent evolutionary branches from an unsampled common ancestor. This complexity increased after we found that the variants from the intermediate specimens (August), which were initially interpreted as co-infection of variants A and B, corresponded to mixtures of variant A with 2 novel intermediate variants. The detection of these new variants allowed us to infer partially the pathway followed in the emergence of variant B. Our findings are consistent with those reported for macaque models (Lin et al., 2014), where different clonal variants in different lesions evolved independently, that is, some finished in a *cul de sac*, whereas others progressed. The finding of different clonal variants in different lesions could lead to a heterogeneous drain of variants into respiratory samples and differential identification of one variant or another.

The variability of the evolutionary routes explored by our strain was also illustrated by the analysis of a further 2 cases of tuberculosis in the population involved in the same transmission cluster as the first study patient. The isolates from these 2 cases shared the same evolutionary branch, although this was independent of that of the intrapatient variants A and B.

Taken together, the SNP data and the subtle differences identified by RFLP and MIRU-VNTR suggest that the strain in question had a higher than expected tendency to microevolve. This is supported not only by its higher than expected accumulation of SNPs, but also by the variations according to IS6110 distribution and by the variations in the number of repetitions for certain VNTR loci. This tendency is not restricted to a specific host, because acquisition of variability was observed in all 3 clustered TB cases analyzed. The most obvious alert is that clusters involving strains of this kind are likely to exceed the similarity thresholds established to define clusters; therefore, related cases could be misinterpreted as unclustered.

The WGS analysis also provided clues about the potential functional significance of some of the above mentioned evolutionary routes followed by the clonal variants. In the case of specific SNPs for variant A, the most remarkable polymorphism is a non-synonymous substitution mapping in the essential gene *mce3R*, a transcriptional repressor of the *mce*3R regulon, which is involved in lipid metabolism and redox reactions (de la Paz Santangelo et al., 2009). The expression of the first gene in the *mce*3 operon, *yrbE3A*, in variant A was lower than in variant B, indicating that the SNP found in variant A leads to higher efficiency of the repressor, which would likely have a functional effect. At some stage in the infection process, more marked repression of this operon might have been advantageous, since both variant A and one of the intermediate variants following a different evolutionary branch shared this SNP.

The *in silico* analysis of the SNPs detected in the clonal variants revealed the involvement of enzymatic, membrane, and cell division proteins. Together with differential expression for variants showing a SNP in *mce*3R and their differences in TNFα production when infecting macrophages (Martín et al., 2011), this involvement indicated the potential functional impact of several of the specific SNPs acquired for each clonal variant.

These findings led us to explore in detail the infective behavior of the clonal variants. Based on a competitive strategy when assaying infectivity in infection models developed in our group (Martín et al., 2011; Navarro et al., 2013) we revealed that clonal variant B was more successful than variant A. This advantageous behavior was observed even when underrepresented (Barczak et al., 2005), and, surprisingly, likely owing to the marked imbalance forced in the co-infection assay, a new variant with a higher fitness emerged, namely variant C, which had not been identified in the clinical specimens. This finding suggested a marked tendency of this strain to microevolve.

In summary, we present an in-depth study of multiple clonal variants which emerged in sequential stages in a patient with recurrent tuberculosis through several independent exploratory branches in the same microevolution event. In addition, other microevolutionary branch leading to different variants was detected in other hosts sharing the same transmission cluster, and finally a novel variant not sampled from the clinical specimens emerged in an infection assay in the laboratory. We also observed differential gene expression and differential infectivity between some of the emerged variants.

These observations emphasize how complex and functional the microevolutionary phenomena in the infection by *M tuberculosis* can be and indicate that some strains are especially prone to microevolution, which could impact on the inference of clusters based on WGS if strict thresholds are applied.

## Ethics statement

The Institutional Animal Care and Use Committee (IACUC) of the Gregorio Marañón General Hospital (ES 280790000087) (Madrid, Spain) reviewed and approved the experimental protocol. The procedures followed were in agreement with the current Spanish Legislation (RD 53/2013), the European Directive 2010/63/UE [which follows the guidelines and recommendations approved by the Federation of Laboratory Animal Science Associations (FELASA)] and the ethical rules which are applied in this center.

## Author contributions

DG, LP-L, JS, IC, MH, YN, and OS: Made substantial contributions to the concept and design of the work through acquisition, analysis and interpretation of data. YN, LP-L, and DG: Drafted the work and provided critical revision for important intellectual content. All: Approved the final version of the article.

### Conflict of interest statement

The authors declare that the research was conducted in the absence of any commercial or financial relationships that could be construed as a potential conflict of interest.
