# Supplementary material for: In-Depth Characterization and Functional Analysis of Clonal Variants in a Mycobacterium tuberculosis Strain Prone to Microevolution
Source: Front Microbiol. 2017 Apr 24;8:694. doi: 10.3389/fmicb.2017.00694 (PMC5403423; doi:10.3389/fmicb.2017.00694)
Supplement: Supplementary file 1 [file SupplementaryTable.docx]

| Fw1201 | 5´-GTCTGCTCGGCGCCAAC-3´ |
| --- | --- |
| Fm1201 | 5´-GTCTGCTCGGCGCCAAA-3´ |
| R1201 | 5´-GTTGGCGTGCAGGTCCTC-3´ |
| Fw1527 | 5´-GATCAACGCACCACTGCC-3´ |
| Fm1527 | 5´-GATCAACGCACCACTGCT-3´ |
| R1527 | 5´-CCATCATGGCTGGTCTCTACT-3´ |
| F1553 | 5´-GATCAACGGCGACCCAAAG-3´ |
| Rw1553 | 5´-TGAACTGCTTGAATGCATCGAG-3´ |
| Rm1553R | 5´-TGAACTGCTTGAATGCATCGAC-3 |
| F1-1963 | 5´-CCCGATCTGAGTCCACAGCATTC-3´ |
| R1w-1963 | 5´-CCTCGTCGCGTGCGGC-3 |
| R1m-1963 | 5´-CCTCGTCGCGTGCGGT-3 |
| F2209 | 5´-GCTCAAACGGTGGTCGCC-3´ |
| Rw2209 | 5´-CCCGATTCGTGTCGGTGC-3´ |
| Rm2209 | 5´-CCCGATTCGTGTCGGTGG-3´ |
| Rv2579Fw | 5´-CGACATGCCGAAACTGTTCA-3´ |
| Rv2579Fm | 5´-CGACATGCCGAAACTGTTCC-3´ |
| Rv2579R | 5´-CCGATTTCCTCTGGGCTGTC-3´ |

Supplementary table: Primers used in the ASO-PCR assays
